# Impaired lung function and bronchodilator responsiveness in former very preterm infants at preschool age: associated factors and clinical implications

**DOI:** 10.3389/fped.2026.1837119

**Published:** 2026-08-05

**Authors:** Michaela Höck, Anna Zschocke, Ulrike Pupp Peglow, Anna Staudt, Carolin Marcher, Maria Schütz, Monika Kofler, Kathrin Gaaß, Barbara Brunner, Ursula Kiechl-Kohlendorfer, Elke Griesmaier

**Affiliations:** 1 Department of Paediatrics II (Neonatology), Medical University of Innsbruck, Innsbruck, Austria; 2 Department of Paediatrics III (Cardiology, Pulmonology, Allergology and Cystic Fibrosis), Medical University of Innsbruck, Innsbruck, Austria

**Keywords:** bronchodilator responsiveness, lung function, preschool age, preterm infants, spirometry

## Abstract

**Background:**

Preterm birth adversely affects pulmonary development. This study aimed to describe lung function and bronchodilator responsiveness in preschool-aged children born very preterm and to explore perinatal and postnatal factors associated with abnormal spirometry.

**Methods:**

We conducted a retrospective cohort study including children born between 2016 and 2019 and admitted to Innsbruck Medical University Hospital with a gestational age below 32 weeks or a birth weight below 1,500 grams. At preschool age, spirometry was performed before and after bronchodilator inhalation.

**Results:**

In total, 107 former preterm infants were assessed at a median age of 5.1 years (median gestational age 29.9 weeks; median birth weight 1,250 grams). Of those, 93 (86.9%) children successfully completed spirometry. Twenty-nine children (31%) showed abnormal spirometry and demonstrated greater bronchodilator responsiveness compared with those with normal spirometry (median change in forced expiratory volume in one second: 15.0% vs. 4.9%, *p* < 0.001). Univariate analyses showed differences between children with normal and abnormal spirometry in the duration of invasive mechanical ventilation (81.6 vs. 23.0 h, *p* = 0.035), postnatal glucocorticoid therapy (24.1% vs. 7.8%, *p* = 0.049), and familial atopic predisposition (55.2% vs. 25.0%, *p* = 0.002). Additionally, abnormal spirometry showed higher rates of respiratory infections (65.5% vs. 18.8%, *p* < 0.001) and increased use of asthma medication (58.6% vs. 34.3%, *p* = 0.027).

**Conclusion:**

In this cohort of children born very preterm, persistent abnormalities in expiratory lung function and increased bronchodilator responsiveness were observed at preschool age. Longer duration of invasive ventilation, glucocorticoid therapy, recurrent respiratory infections, and familial atopic predisposition were associated with poorer pulmonary outcomes.

## Introduction

Advancements in obstetric and neonatal care have significantly improved survival and reduced morbidity among preterm infants (1). As a result, an increasing number of individuals born preterm are reaching childhood and adulthood, highlighting the need to better understand long-term respiratory outcomes after preterm birth.

Infants born very preterm, particularly those with a history of bronchopulmonary dysplasia (BPD), are at increased risk for chronic respiratory morbidity, which may persist into later life (2–4). These outcomes are increasingly recognized and may encompass distinct prematurity-associated lung disease phenotypes, including impaired alveolar development, fixed airway obstruction, and phenotypes characterized by bronchial hyperresponsiveness and asthma-like features. Recognition of this phenotypic heterogeneity is increasingly important, as the underlying mechanisms and risk profiles may differ between these patterns of lung impairment.

Previous studies have shown that preterm infants are at higher risk of recurrent respiratory infections, airway obstruction, and bronchial hyperresponsiveness (5, 6). They are also more likely to require bronchodilator therapy during infancy and childhood, particularly those born at lower gestational ages, with lower birth weight, or with BPD (7). By school age, over 50% of children born extremely preterm exhibit abnormal baseline spirometry and up to 30% show bronchial hyperresponsiveness (8). These findings underscore the importance of structured and longitudinal respiratory follow-up in this vulnerable population (9).

In 2022, we implemented a standardized routine respiratory follow-up program for preterm infants born at less than 32 weeks of gestation or with a birth weight below 1,500 grams. This program includes spirometry and bronchodilator responsiveness testing as part of a routine clinical follow-up program at preschool age. In our previous study we focused on the evaluation of feasibility and acceptance of preschool age lung function test, including forced expiratory maneuvers and bronchodilator responsiveness testing (10). The aim of the present study was to describe lung function and bronchodilator responsiveness in preschool-aged children born very preterm and to explore perinatal and postnatal factors associated with impaired spirometry within this cohort.

## Methods

### Participants

This retrospective cohort study of prospectively collected data was conducted at Innsbruck Medical University Hospital, which provides the only neonatal intensive care unit in Tyrol, a state in western Austria with a population of 777,000 and approximately 7,000 live births annually. The study included preschool-aged children (5–6 years old) born between 2016 and 2019 at less than 32 weeks of gestation or with a birth weight below 1,500 g.

### Neonatal characteristics

Perinatal and neonatal data were collected during hospital stay, including factors that could influence pulmonary function, such as very low birth weight (11), surfactant administration [less invasive surfactant administration (LISA) or via an endotracheal tube] (12), duration of mechanical ventilation, continuous positive airway pressure and supplemental oxygen. BPD was defined according to National Institutes of Health consensus criteria, including oxygen requirement for >28 days and severity classification according to respiratory support at 36 weeks postmenstrual age (1, 13). We also included the need for postnatal glucocorticoid therapy, hemodynamically significant ductus arteriosus (clinical signs of pulmonary overcirculation or systemic hypoperfusion, or echocardiographic indicators, such as an internal diameter of duct > 1.5 mm or left atrium-to-aortic root ratio > 1.6) (14), necrotizing enterocolitis according to Bell's criteria (15), early (<72 h) and late (>72 h) onset sepsis (clinical signs of generalized infection and antibiotic therapy for >5 days), cytomegalovirus or ureaplasma infections, intracranial hemorrhage according to Papile et al. (16), retinopathy of prematurity (17), nutrition (parenteral feeding, milk fortifiers) (18), and indication for respiratory syncytial virus prevention.

As part of our routine pulmonary follow-up program, we performed a clinical examination, obtained a medical history (including pulmonary and atopic diseases), and perfomed spirometry and bronchodilator responsiveness testing.

### Clinical examination

As described in detail in our previous work (10), the clinical examination comprised anthropometric measurements, including calculation of body mass index, as well as assessment of heart rate and oxygen saturation by pulse oximetry. Neurodevelopmental follow-up additionally included evaluation of cognitive outcome, with assessment of the intelligence quotient. Together, these measurements provided baseline data on the physical status of the participants, cardiorespiratory parameters, and cognitive development.

### Medical history survey

A detailed medical survey, based on the International Study of Asthma and Allergies in Childhood questionnaire (19), was conducted, focusing on respiratory morbidity after the neonatal period. Frequent infections were defined as recurrent respiratory tract infections according to established pediatric criteria: six or more respiratory tract infections per year, upper respiratory tract infections occurring at least monthly during the cold season, or three or more lower respiratory tract infections per year. Respiratory morbidity was defined as parent-reported chronic or recurrent respiratory symptoms, such as wheeze, chronic cough, or dyspnea, that prompted medical attention (physician visit, emergency department attendance, hospitalization, or prescription treatment) during early childhood. Clinically relevant respiratory morbidity at study visit was defined as recurrent wheeze, chronic cough, exercise-related respiratory limitation, physician-diagnosed asthma, or regular use of inhaled respiratory medication. Use of asthma medication was defined as parent-reported treatment with inhaled bronchodilators and/or inhaled corticosteroids during early childhood. Because no standardized allergy testing was performed, familial atopic predisposition was assessed by parental report rather than by confirmed atopic disease in the participating children. Information on current and past smoking habits (including during pregnancy) and on exposure to pets was collected.

### Spirometry

Spirometry was conducted using a Jaeger MasterScope spirometer (VIASYS Healthcare, Carl Reiner, Vienna) and performed in accordance with the American Thoracic Society and European Respiratory Society recommendations for preschool children. Flow–volume curves were visually assessed for quality: only technically acceptable maneuvers without major artefacts (e.g., cough, glottic closure, or air leak) were considered. Children were allowed up to ten attempts to achieve a minimum of three reproducible measurements. The best technically acceptable manoeuvre was used for analysis.

The following spirometry parameters were measured: forced expiratory volume in one second (FEV₁), forced vital capacity (FVC), Tiffeneau index (FEV₁/FVC), peak expiratory flow (PEF), forced expiratory flow at 25%, 50%, and 75% of FVC (FEF₂₅, FEF₅₀, and FEF₇₅), and mean forced expiratory flow between 25% and 75% of FVC (MFEF₂₅–₇₅). Results were expressed as z-scores using Global Lung Function Initiative (GLI 2012) reference equations, which are validated for European children (LUNOKID study). Spirometric abnormalities were defined as values below the lower limit of normal, corresponding to z-scores < −1.64 for FVC, FEV₁, and/or PEF, in accordance with international reference standards (20–22).

### Bronchodilator responsiveness test

Bronchodilator responsiveness may provide clinically relevant information on reversible airflow limitation in preschool-aged children born preterm. All spirometry assessments were performed both before and after bronchodilator administration (23). After baseline spirometry, each child received four doses of salbutamol (0.1 mg/dose) via a spacer with a mouthpiece. Following a 10-minute waiting period, children were allowed up to ten attempts to achieve a minimum of three reproducible measurements, and the best technically acceptable manoeuvre was used for analysis. A positive bronchodilator response was defined as an increase in FEV₁ of ≥ 12% from baseline (24).

### Statistical analysis

Statistical analyses were performed using SPSS Statistics (IBM Corp., Armonk, NY, USA). Continuous variables were assessed for normality using visual inspection and the Shapiro–Wilk test. Normally distributed variables are presented as mean ± standard deviation (SD) and were compared using the Student's t-test. Non-normally distributed variables are presented as median and interquartile range (IQR) and were analyzed using the Mann–Whitney U test. Categorical variables are reported as frequencies and percentages and were compared using the chi-square test or Fisher's exact test, as appropriate.

Primary analyses focused on comparisons between children with normal pulmonary outcomes (normal group) and those with pathological pulmonary findings (pathological group). Additional analyses examining associations between pulmonary function and neonatal or familial factors, including surfactant administration mode, milk fortifier type, recurrent infections, and familial atopic predisposition, were considered exploratory subgroup analyses and should be interpreted as hypothesis-generating.

Given the limited number of children with pathological pulmonary outcome (*n* = 29), multivariable regression analyses were considered to carry a high risk of overfitting and model instability. Analyses were therefore performed primarily in an exploratory, univariable manner, and no formal predictor selection procedure or multivariable adjustment model was applied.

A two-sided *p* value <0.05 was considered statistically significant.

### Compliance with ethical standards

This study was approved by the Ethics Committee of the Medical University of Innsbruck (1013/2023) and was conducted in accordance with the principles of the Declaration of Helsinki (2024 revision).

## Results

### Characteristics of the study population

A total of 121 preterm infants born before 32 weeks of gestation or with a birth weight below 1,500 g were eligible for inclusion. Five children (4.1%) were excluded because of severe neurological impairment precluding reliable pulmonary function testing, and nine children (7.4%) were lost to follow-up. The final study cohort therefore comprised 107 children assessed at preschool age. Spirometry was successfully completed in 93 participants (86.9%); the remaining 14 children (13.1%) were excluded because of insufficient spirometry quality.

Participants were classified into two groups according to a predefined clinical follow-up protocol established in our previously published study (10): children with normal pulmonary function and no clinically relevant respiratory symptoms (normal group), and children with pathological pulmonary findings (pathological group). Group allocation to the pathological group was primarily based on spirometric results.

**Figure 1:**
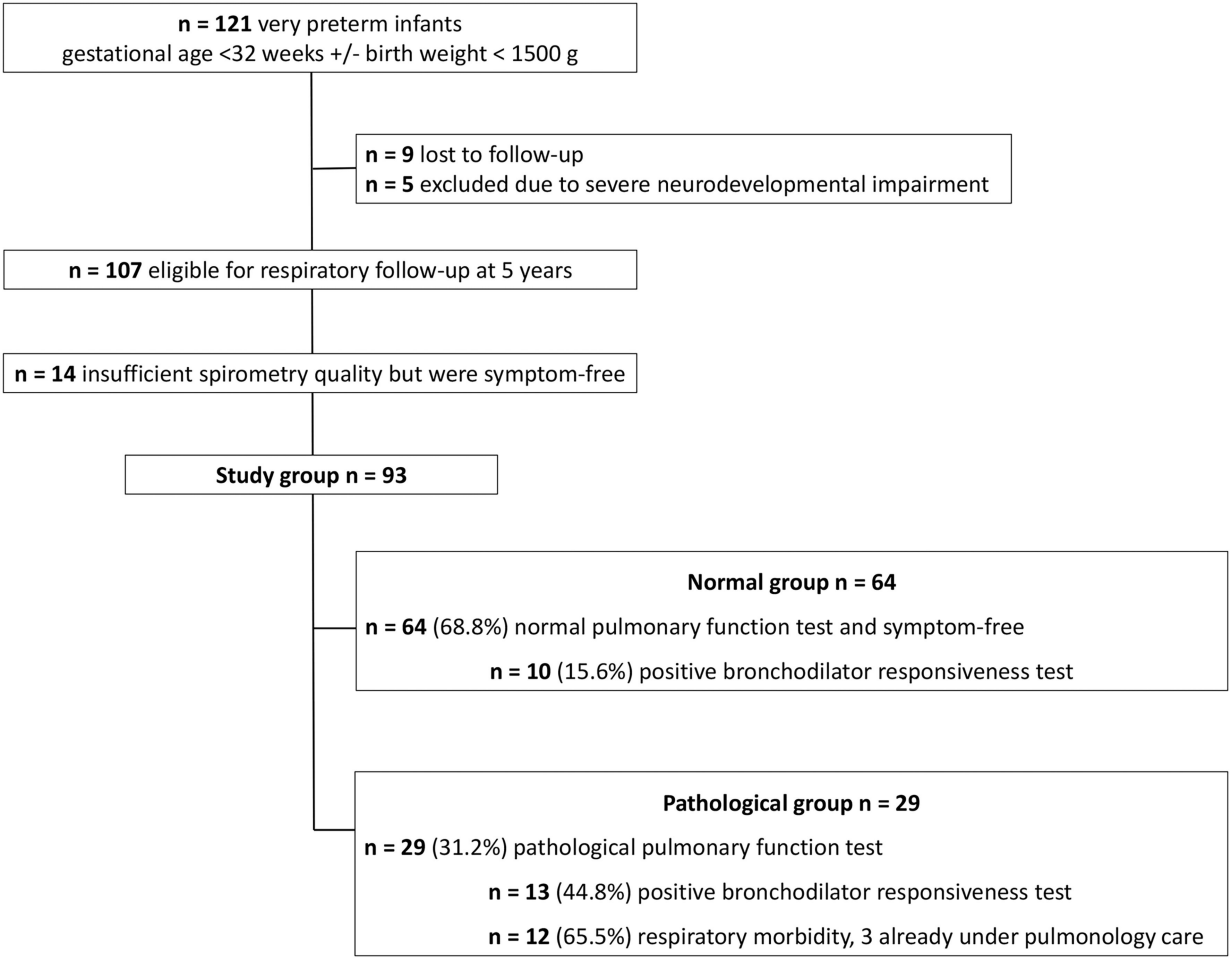
Participant flow diagram.

A detailed flow diagram is shown in Figure 1.

The children had a median age of 5.1 years at assessment (IQR 5.0–5.3), 59.8% were male, and all were of European ancestry. Anthropometric parameters, oxygen saturation, heart rate, and intelligence quotient did not differ significantly between the groups. All children were clinically stable at the time of assessment and showed no signs of acute lower respiratory tract infection. Mild upper respiratory tract symptoms consistent with rhinitis were present in nine children (8.4%), and were not classified as clinically relevant respiratory symptoms.

Children in the pathological group showed a significantly higher frequency of frequent infections than those in the normal group (65.5% vs. 18.8%; *p* < 0.001). The use of asthma medication was also more frequent in the pathological group (58.6% vs. 34.4%; *p* = 0.027), as was a positive familial atopic predisposition (55.2% vs. 25.0%; *p* = 0.002). Although 15 (23.4%) children in the normal group had experienced recurrent respiratory morbidity during early childhood, they were clinically stable at the preschool follow-up. In contrast, 12 (41.4%) children in the pathological group continued to have clinically relevant respiratory morbidity at 5 years of age.

We observed no significant difference between groups with respect to maternal smoking during pregnancy (0% vs. 4.7%; *p* = 0.221), postnatal tobacco exposure (13.8% vs. 20.3%; *p* = 0.493), or pet ownership (27.6% vs. 18.8%; *p* = 0.236).

Characteristics at the study visit and parent-reported medical history are summarized in Table 1.

**Table 1:** Characteristics at the study visit and parent-reported medical history.

Characteristics	normal group (*n* = 64)	pathological group (*n* = 29)	*p* value
Characteristics			
Age [years]; median (IQR)	5.1 (5.0−5.2)	5.1 (5.0−5.2)	0.594
Height [cm]; mean (SD)	109.0 (5.6)	109.2 (4.3)	0.838
Weight [kg]; mean (SD)	17.5 (3.2)	17.4 (2.5)	0.880
Body mass index [kg/m^2^]; mean (SD)	14.6 (1.7)	14.5 (1.2)	0.743
Oxygen saturation [%]; median (IQR)	99 (98–100)	99 (99–100)	0.745
Heart rate [bpm]; mean (SD)	97.9 (13.7)	97.1 (10.8)	0.777
Intelligence quotient; mean (SD)	92.8 (13.8)	94.7 (20.1)	0.583
Medical history			
Frequent infections; *n* (%)	12 (18.8)	19 (65.5)	**<0**.**001**
Respiratory morbidity; *n* (%)	15 (23.4)	12 (41.4)	0.143
Asthma medication; *n* (%)	22 (34.4)	17 (58.6)	**0**.**027**
Familial atopic predisposition; *n* (%)	16 (25.0)	16 (55.2)	**0**.**002**
Smoking in pregnancy; *n* (%)	3 (4.7)	0 (0)	0.221
Smoking exposure; *n* (%)	13 (20.3)	4 (13.8)	0.493
Pet ownership; *n* (%)	12 (18.8)	8 (27.6)	0.236

Data are presented as mean (SD), median (IQR) or *n* (%). Bold values indicate statistical significance (*p* < 0.05). IQR, interquartile range; SD, standard deviation; bpm, beats per minute.

As reported previously (10), no significant differences were observed between groups with respect to neonatal baseline characteristics. The median gestational age of the cohort was 29.9 weeks (IQR 28.1–31.1), and the mean birth weight was 1250.5 g (±355.6). Antenatal corticosteroids were documented in 93.5% of infants, and 74.8% received surfactant therapy, of whom 49.5% were treated with LISA.

Children in the pathological group required a significantly longer duration of invasive mechanical ventilation than those in the normal group (81.6 ± 195.8 h vs. 23.0 ± 59.5 h; *p* = 0.035). Postnatal glucocorticoid therapy for BPD was administered more frequently in the pathological group (24.1% vs. 7.8%; *p* = 0.049). The duration of supplemental oxygen therapy tended to be longer in the pathological group (22.5 ± 29.2 days vs. 11.8 ± 19.4 days; *p* = 0.056). No statistically significant differences were found between groups in the incidence of BPD, necrotizing enterocolitis, early- or late-onset sepsis, intraventricular hemorrhage, or retinopathy of prematurity.

Detailed neonatal characteristics are summarized in Table 2.

**Table 2:** Detailed neonatal characteristics.

Neonatal Characteristics	normal group (*n* = 64)	pathological group (*n* = 29)	Insufficient spirometry quality (*n* = 14)	*p* value
Gestational age [weeks]; median (IQR)	29.9 (28.3–31.1)	30.0 (26.4–31.2)	28.9 (25.4–31.0)	0.651
Birth weight [grams]; mean (SD)	1304.4 (±361.6)	1204.0 (±345.2)	1100.8 (±311.8)	0.212
Small for gestational age; *n* (%)	12 (18.8)	3 (10.3)	4 (28.6)	0.307
Male; *n* (%)	36 (56.3)	19 (65.5)	9 (64.3)	0.400
Apgar score 1 min; median (IQR)	7 (6–8)	7 (6–8)	6 (5–7)	0.355
Apgar score 5 min; median (IQR)	8 (8–9)	8 (8–9)	8 (7–9)	0.488
Apgar score 10 min; median (IQR)	9 (9–9)	9 (9–9)	9 (8–9)	0.614
Umbilical cord arterial pH; mean (SD)	7.33 (±0.07)	7.34 (±0.07)	7.33 (±0.05)	0.570
Umbilical cord venous pH; mean (SD)	7.31 (±0.13)	7.39 (±0.05)	-	0.302
Antenatal corticosteroids; *n* (%)	53 (82.8)	27 (93.1)	13 (92.9)	0.629
Complete; *n* (%)	41 (64.1)	22 (75.9)	9 (64.3)	-
Incomplete; *n* (%)	12 (18.8)	5 (17.2)	4 (28.6)	-
Surfactant; *n* (%)	47 (73.4)	21 (72.4)	12 (85.7)	0.842
Less invasive surfactant administration; *n* (%)	33 (51.6)	11 (37.9)	10 (71.4)	0.155
Intubation; *n* (%)	14 (21.9)	10 (34.5)	2 (14.3)	-
Duration of invasive ventilation [hours]; mean (SD)	23.0 (±59.5)	81.6 (±195.8)	140.6 (±273.6)	**0**.**035**
Duration of CPAP [days]; mean (SD)	16.3 (±17.7)	21.2 (±21.5)	19.1 (±22.0)	0.271
Duration of supplemental FiO_2_ [days]; mean (SD)	11.8 (±19.4)	22.5 (±29.2)	33.1 (±43.6)	0.056
Bronchopulmonary dysplasia; *n* (%)	9 (14.1)	8 (27.6)	5 (35.7)	0.143
BPD mild; *n* (%)	8 (12.5)	5 (17.2)	2 (14.3)	0.373
BPD moderate; *n* (%)	1 (1.6)	2 (6.9)	1 (7.1)	-
BPD severe; *n* (%)	0 (0)	1 (3.4)	2 (14.3)	-
BPD glucocorticoid therapy; *n* (%)	5 (7.8)	7 (24.1)	5 (35.7)	**0**.**049**
Parenteral feeding [days]; mean (SD)	14.7 (±10.2)	17.2 (±18.8)	28.3 (±29.0)	0.433
Milk fortifier; *n* (%)	51 (79.7)	24 (82.8)	12 (85.7)	0.682
Human-based; *n* (%)	11 (17.2)	3 (10.3)	5 (35.7)	-
Bovine-based; *n* (%)	40 (62.5)	21 (72.4)	7 (50.0)	-
Patent ductus arteriosus; *n* (%)	11 (17.2)	4 (13.8)	3 (21.4)	0.638
Necrotizing enterocolitis; *n* (%)	2 (3.1)	1 (3.4)	1 (7.1)	0.965
Sepsis; *n* (%)	13 (20.3)	7 (24.1)	4 (28.6)	0.346
Early-onset sepsis; *n* (%)	8 (12.5)	2 (6.9)	2 (14.3)	-
Late-onset sepsis; *n* (%)	5 (7.8)	5 (17.2)	2 (14.3)	-
Cytomegalovirus; *n* (%)	1 (1.6)	2 (6.9)	2 (14.3)	0.171
Ureaplasma; *n* (%)	0 (0)	1 (3.4)	2 (14.3)	0.065
Intraventricular hemorrhage; *n* (%)	8 (12.5)	5 (17.2)	2 (14.3)	0.577
Retinopathy of prematurity; *n* (%)	11 (17.2)	7 (24.1)	6 (42.9)	0.749
ROP I; *n* (%)	6 (9.4)	4 (13.8)	2 (14.3)	-
ROP II; *n* (%)	4 (6.3)	3 (10.3)	3 (21.4)	-
ROP III; *n* (%)	1 (1.6)	0 (0)	1 (7.1)	-
Discharge [days]; median (IQR)	45.0 (36.0–62.0)	52.0 (33.0–76.0)	65.9 (44.5–76.3)	0.357
RSV prophylaxis; *n* (%)	43 (67.2)	22 (75.9)	11 (78.6)	0.389

Continuous variables are presented as mean ± SD or median (IQR), as appropriate; categorical variables are presented as *n* (%). *p* values refer to the comparison between the normal and pathological groups; the group with insufficient spirometry quality (*n* = 14) is shown for descriptive purposes only and was not included in the statistical comparison. Bold values indicate statistical significance (*p* < 0.05). BPD, bronchopulmonary dysplasia; CPAP, continuous positive airway pressure; FiO₂, fraction of inspired oxygen; IQR, interquartile range; ROP, retinopathy of prematurity; RSV, respiratory syncytial virus; SD, standard deviation.

### Spirometry results

Children in the pathological group showed significantly lower spirometric values than the normal group. Median FVC was 79.9% (IQR 69.3–88.4) vs. 94.8% (IQR 87.0–104.2; *p* < 0.001), and corresponding FVC z-scores of −1.5 (IQR −2.3 to −0.9) vs. −0.4 (IQR −1.0 to 0.3; *p* < 0.001). FEV₁ was likewise significantly reduced (77.3% predicted, IQR 72.6–83.7 vs. 95.5% predicted, IQR 89.4–106.5; *p* < 0.001), as were FEV₁ z-scores (−1.7, IQR −2.1 to −1.3 vs. −0.3, IQR −0.8 to 0.6; *p* < 0.001). Additional indices reflecting mid- and small-airway function –PEF, MFEF, and forced expiratory flow values—were also significantly lower in the pathological group (all *p* < 0.001 except FEF_75_, *p* = 0.004), and the corresponding z-score analyses confirmed these findings. No significant difference in the FEV₁/FVC ratio was observed between the groups (*p* = 0.059).

### Bronchodilator responsiveness testing

The magnitude of bronchodilator-induced change in FEV₁ was significantly greater in the pathological group than in the normal group (median 14.9%, IQR 7.6–23.5, vs. 4.9%, IQR 0.5–10.3; *p* < 0.001). Accordingly, a positive bronchodilator response, defined as an increase in FEV₁ of ≥ 12% from baseline, was observed significantly more often in the pathological group than in the normal group (44.8% vs. 15.6%; *p* < 0.001). The number of spirometry maneuvers required after bronchodilator administration was comparable between the groups (median 6.5, IQR 6–8 vs. 7, IQR 5–9; *p* = 0.719).

Detailed spirometric results are summarized in Table 3.

**Table 3:** Results from spirometry and bronchodilator responsiveness testing.

Spirometry	normal group (*n* = 64)	pathological group (*n* = 29)	*p* value
FVC; median (IQR)	94.8 (87.0–104.2)	79.9 (69.3–88.4)	**<0.001**
FVC z-score; median (IQR)	−0.4 (−1.0 to 0.3)	−1.5 (−2.3 to −0.9)	**<0.001**
FEV₁; median (IQR)	95.5 (89.4–106.5)	77.3 (72.6–83.7)	**<0.001**
FEV₁ z-score; median (IQR)	−0.3 (−0.8 to 0.6)	−1.7 (−2.1 to −1.3)	**<0.001**
FEV₁/FVC; median (IQR)	92.9 (88.5–97.8)	86.8 (80.6–98.9)	0.059
FEV₁/FVC z-score; median (IQR)	−0.1 (−0.6 to 1.0)	−0.5 (−1.8 to 1.4)	0.242
PEF; median (IQR)	92.2 (80.8–103.2)	73.7 (64.8–86−6)	**<0.001**
PEF z-score; median (IQR)	−0.6 (−1.4 to 0.2)	−1.9 (−2.5 to −1.0)	**<0.001**
MFEF; median (IQR)	89.9 (75.4–108.4)	70.3 (51.9–87.1)	**<0.001**
MFEF z-score; median (IQR)	−0.4 (−1.0 to 0.3)	−1.2 (−2.1 to −0.5)	**<0.001**
FEF_25_; median (IQR)	93.2 (81.3–102.0)	72.0 (62.0–83.0)	**<0.001**
FEF_25_ z-score; median (IQR)	−0.5 (−1.4 to 0.1)	−2.0 (−2.7 to −1.2)	**<0.001**
FEF_50_; median (IQR)	99.7 (84.7–117.9)	76.9 (59.7–92.1)	**<0.001**
FEF_50_ z-score; median (IQR)	−0.02 (−0.6 to 0.7)	−0.9 (−1.7 to −0.3)	**<0.001**
FEF_75_; median (IQR)	100.1 (79.2–117.6)	73.0 (50.8–109.7)	**0.002**
FEF_75_ z-score; median (IQR)	0.0 (−0.7 to 0.5)	−098 (−1.8 to 0.3)	**0.002**
Attempts before BRT; median (IQR)	8 (6–10)	8 (6–9.5)	0.452
Bronchodilator responsiveness test			
Positive BRT (FEV_1_ ≥ 12%); *n* (%)	10 (15.6)	13 (44.8)	**<0.001**
Change in FEV₁; median (IQR)	4.9 (0.5–10.3)	14.9 (7.6–23.5)	**<0.001**
Attempts after BRT; median (IQR)	7 (5–9)	6.5 (6–8)	0.719

Data are presented as median (IQR), except for categorical variables, which are presented as *n* (%). *p* values refer to the comparison between the normal and pathological group. Bold values indicate statistical significance (*p* < 0.05). BRT, bronchodilator responsiveness test; FEF₂₅, FEF₅₀, FEF₇₅, forced expiratory flow at 25%, 50%, and 75% of FVC; FEV₁, forced expiratory volume in one second; FVC, forced vital capacity; MFEF, mean forced expiratory flow between 25% and 75% of FVC; PEF, peak expiratory flow; IQR, interquartile range.

### Exploratory subgroup analyses

#### Surfactant administration

Children who received surfactant via less invasive surfactant administration (LISA) demonstrated significantly higher FVC (*p* = 0.012) and FVC z-scores (*p* = 0.020) than those treated via endotracheal surfactant administration. No significant differences were observed for FEV₁, FEV₁/FVC ratio, PEF, MFEF, FEF25–75, bronchodilator responsiveness, or the number of spirometry attempts before and after bronchodilator administration (all *p* > 0.05).

#### Milk fortifier

Children who received human-based milk fortifier (Prolacta) demonstrated significantly higher FVC (*p* = 0.043), FEV₁ (*p* = 0.005), PEF (*p* = 0.031), MFEF (*p* = 0.009), and expiratory flow parameters (FEF25, *p* = 0.043; FEF50, *p* = 0.016; FEF75, *p* = 0.020) than those receiving bovine-based fortifier, whereas no significant differences were observed for the FEV₁/FVC ratio or bronchodilator responsiveness outcomes.

#### Familial atopic predisposition

Children with a positive familial atopic predisposition had significantly lower FVC (*p* < 0.001), FVC z-scores (*p* = 0.002), FEV₁ (*p* = 0.003), and FEV₁ z-scores (*p* = 0.003) compared with children without familial atopy. No significant differences were observed for the FEV₁/FVC ratio, expiratory flow parameters, or bronchodilator responsiveness outcomes (all *p* > 0.05).

#### Frequent infections

Children with frequent respiratory infections had significantly lower FEV₁ (*p* = 0.010), FEV₁ z-scores (*p* = 0.007), MFEF (*p* = 0.017), MFEF z-scores (*p* = 0.018), and mid-to-late expiratory flow parameters (FEF25–75, *p* = 0.013–0.029) compared with children without frequent infections. FVC showed a borderline reduction (*p* = 0.047), whereas no significant differences were observed for the FEV₁/FVC ratio, PEF, or bronchodilator responsiveness outcomes (all *p* > 0.05).

## Discussion

A growing body of evidence highlights the negative impact of prematurity on long-term pulmonary health, shifting the research focus from short-term neonatal morbidity towards lifelong respiratory outcomes. Early identification of associated factors for pulmonary impairment is therefore essential to guide preventive and therapeutic strategies. The aim of our study was to evaluate pulmonary function at preschool age in children born very preterm and to explore potential perinatal and neonatal factors associated with abnormal spirometry.

### Pulmonary function after preterm birth

In this population-based Tyrolean cohort, approximately 30% of very preterm children demonstrated impaired lung function at preschool age, with or without bronchodilator responsiveness. Spirometric parameters such as FVC, FEV₁, PEF, and FEF were significantly reduced, while the FEV₁/FVC ratio remained unaffected. This pattern may reflect altered alveolar development, dysanaptic airway growth, or disrupted postnatal lung development—recognized hallmarks of prematurity—although reduced FVC should be interpreted cautiously, as body plethysmography was not available and therefore true restrictive impairment cannot be distinguished from pseudo-restriction due to air trapping. These findings are in line with previous studies showing that preterm birth adversely affects both large and small airway development and contributes to persistent respiratory vulnerability (25, 26).

### Bronchodilator responsiveness

Bronchodilator responsiveness reflects reversible airflow limitation following administration of a β2-agonist. It is quantified by the change in spirometric parameters after bronchodilation and may indicate altered airway development and increased susceptibility to airway dysfunction in children and adolescents born preterm. Preterm birth interrupts late-gestational airway growth and exposes the immature lung to inflammatory and mechanical stressors (27), resulting in relatively smaller airways, increased smooth-muscle reactivity (28), and impaired epithelial repair (29). Consequently, former preterm infants—both with and without BPD—may demonstrate increased bronchodilator responsiveness and, in studies using formal challenge testing, also increased airway reactivity compared with term-born peers. This pattern has been described as part of an asthma-like phenotype characterized by recurrent wheeze and variable airflow limitation during childhood. Bronchodilator responsiveness may reflect increased airway lability in this population and are clinically relevant as they contribute to persistent respiratory symptoms, increased use of asthma medications, and may influence long-term lung function trajectories into adolescence and adulthood (30, 31).

### Associated factors for impaired lung function

Several perinatal and neonatal factors were associated with abnormal lung function. In exploratory univariable analyses, longer durations of invasive ventilation correlated with reduced pulmonary volumes. Prolonged invasive ventilation is widely recognized as one of the strongest risk factors for long-term respiratory impairment after preterm birth. This finding is consistent with Boven et al. (32), who demonstrated that invasive ventilation is associated with poorer long-term respiratory outcomes, suggesting that cumulative ventilator exposure contributes to structural lung injury.

The observed association may indicate a clinically relevant impact of invasive ventilation and underscores the potential benefit of non-invasive support strategies such as LISA, which was associated with higher FVC values in our cohort; however, this association should be interpreted cautiously, as infants selected for LISA may have had lower baseline respiratory severity.

Glucocorticoid therapy was more common among children with impaired lung function, reflecting its use in severe neonatal respiratory disease and BPD. Prior studies confirm an association between glucocorticoid therapy, BPD severity, and long-term pulmonary dysfunction (7, 8). Interestingly, despite more frequent use of invasive ventilation and glucocorticoids, the incidence of BPD itself did not differ significantly between groups.

Nutritional factors also appeared relevant: children who received human milk fortifier had higher lung function values across multiple spirometric parameters, in line with previous research suggesting beneficial effects of human milk on lung growth and BPD prevention (33). However, these nutritional analyses were exploratory and may have been influenced by clinical selection factors and residual confounding.

### Role of atopy and genetic predisposition

Children with a positive familial atopic predisposition demonstrated lower spirometric values and increased respiratory morbidity, supporting the hypothesis that genetic susceptibility contributes to long-term pulmonary vulnerability after preterm birth. Emerging evidence further supports a genetic contribution to impaired lung development after preterm birth. In particular, polygenic risk for chronic obstructive pulmonary disease has been shown to modify lung function trajectories in individuals born preterm (34). Our findings are in agreement with Guimarães et al., who demonstrated higher rates of respiratory morbidity and atopy in former preterm children, irrespective of BPD status (35). Similarly, Siltanen et al. reported that atopy in preterm-born children was associated with increased respiratory symptoms and impaired lung function (36). Conversely, Greenough et al. (37) observed that chronic lung disease was not the primary determinant of later morbidity, suggesting that multifactorial interactions—including atopy, infections, and environmental exposures—shape long-term respiratory outcomes. Taken together, these data underline the importance of considering both genetic and environmental factors when assessing pulmonary health in preterm-born populations.

### Clinical implications and strengths

Our study emphasizes that respiratory vulnerability persists into preschool age in a substantial proportion of preterm-born children. The increased frequency of infections and asthma medication use in those with impaired lung function points towards ongoing airway dysfunction and susceptibility to chronic respiratory morbidity. Early lung function testing at preschool age may therefore provide a clinically relevant opportunity to identify high-risk children and initiate preventive strategies before lifestyle factors, such as active smoking, become confounding.

A major strength of this study is the comprehensive assessment of lung function in a well-defined cohort with favorable environmental conditions—annual air quality reports in Tyrol during the study period reported pollutant levels well below regulatory thresholds (38). Additionally, the low loss-to-follow-up rate enhances the validity and robustness of our findings.

### Limitations and future directions

Spirometry in preschool-aged children is technically challenging, and not all measurements may fully satisfy ATS/ERS quality criteria. However, experienced pediatric pulmonologists assessed the overall interpretability of lung function examinations in conjunction with clinical findings, reflecting real-world clinical practice. This approach may introduce a degree of subjectivity and potential variability in the classification of pulmonary outcomes. Children unable to perform reliable spirometry because of neurological impairment were excluded. As these children may represent a subgroup with greater overall morbidity, selection bias and potential underestimation of respiratory impairment cannot be excluded.

Several additional subgroup analyses, including those evaluating surfactant administration mode, milk fortifier type, recurrent infections, and familial atopic predisposition, should be interpreted cautiously. These analyses were exploratory in nature and based on a modest sample size. Furthermore, they are susceptible to residual confounding and confounding by indication. For example, infants requiring endotracheal surfactant administration may have represented a subgroup with greater initial respiratory disease severity compared with infants treated via LISA. Similarly, nutritional management strategies may have been influenced by clinical characteristics not fully captured in the present analysis. Consequently, these findings should be regarded as hypothesis-generating and require confirmation in larger prospective studies with adequately powered multivariable analyses.

Another important limitation is the absence of body plethysmography and direct lung volume measurements. Consequently, true restrictive impairment could not be reliably distinguished from pseudo-restriction due to air trapping and obstructive airway disease. Future studies incorporating lung volume assessment, diffusion capacity testing, and longitudinal follow-up into adolescence and adulthood are needed to better characterize the long-term pulmonary phenotype after very preterm birth.

## Conclusion

In this cohort of children born very preterm, persistent abnormalities in expiratory lung function and increased bronchodilator responsiveness were observed at preschool age. Longer duration of invasive ventilation, glucocorticoid therapy, recurrent respiratory infections, and familial atopic predisposition were associated with poorer pulmonary outcomes. Early pulmonary follow-up may help identify children at risk for persistent respiratory morbidity. Future studies incorporating genetic profiling, lung volume measurements, and long-term follow-up are needed to better elucidate the complex interplay of biological, environmental, and clinical factors shaping lifelong respiratory health after preterm birth.

## Data Availability

The raw data supporting the conclusions of this article will be made available by the authors, without undue reservation.
